# Wastewater Based Epidemiology Perspective as a Faster Protocol for Detecting Coronavirus RNA in Human Populations: A Review with Specific Reference to SARS-CoV-2 Virus

**DOI:** 10.3390/pathogens10081008

**Published:** 2021-08-10

**Authors:** Milad Mousazadeh, Razieh Ashoori, Biswaranjan Paital, Işık Kabdaşlı, Zacharias Frontistis, Marjan Hashemi, Miguel A. Sandoval, Samendra Sherchan, Kabita Das, Mohammad Mahdi Emamjomeh

**Affiliations:** 1 Student Research Committee, Qazvin University of Medical Sciences, Qazvin, Iran; m.milad199393@gmail.com; 2 Department of Environmental Health Engineering, School of Health, Qazvin University of Medical Sciences, Qazvin, Iran; 3 Department of Environmental Health Engineering, School of Health, Shiraz University of Medical Sciences, Shiraz, Iran; ra.ashoori90@gmail.com; 4 Redox Regulation Laboratory, College of Basic Science and Humanities, Odisha University of Agriculture and Technology, Bhubaneswar 751003, India; biswaranjanpaital@gmail.com; 5 Environmental Engineering Department, Civil Engineering Faculty, Ayazağa Campus, İstanbul Technical University, İstanbul 34469, Turkey; kabdasli@itu.edu.tr; 6 Department of Chemical Engineering, University of Western Macedonia, 50132 Kozani, Greece; zfrontistis@uowm.gr; 7 Environmental and Occupational Hazards Control Research Center, Shahid Beheshti University of Medical Sciences, Tehran, Iran; hashemimarjan18@gmail.com; 8 Laboratorio de Electroquímica Medio Ambiental LEQMA, Departamento de Química de los Materiales, Facultad de Química y Biología, Universidad de Santiago de Chile USACH, Casilla 40, Correo 33, Santiago 9170022, Chile; miguel.sandoval@usach.cl; 9 Departamento de Ingeniería Química, División de Ciencias Naturales y Exactas, Universidad de Guanajuato, Guanajuato 36050, Mexico; 10 School of Public Health and Tropical Medicine, Tulane University, New Orleans, LA 7011, USA; sshercha@tulane.edu; 11 Department of Philosophy, Utkal University, Bhubaneswar 751004, India; Kabitajnuphilosophy@gmail.com; 12 Social Determinants of Health Research Center, Research Institute for Prevention of Non-Communicable Diseases, Qazvin University of Medical Sciences, Qazvin, Iran

**Keywords:** wastewater-based epidemiology, SARS-CoV-2, COVID-19, coronavirus, detection and quantification protocols

## Abstract

Wastewater-based epidemiology (WBE) has a long history of identifying a variety of viruses from poliovirus to coronaviruses, including novel Severe Acute Respiratory Syndrome Coronavirus 2 (SARS-CoV-2). The presence and detection of SARS-CoV-2 in human feces and its passage into the water bodies are significant public health challenges. Hence, the hot issue of WBE of SARS-CoV-2 in the coronavirus respiratory disease (COVID-19) pandemic is a matter of utmost importance (e.g., SARS-CoV-1). The present review discusses the background, state of the art, actual status, and prospects of WBE, as well as the detection and quantification protocols of SARS-CoV-2 in wastewater. The SARS-CoV-2 detection studies have been performed in different water matrixes such as influent and effluent of wastewater treatment plants, suburban pumping stations, hospital wastewater, and sewer networks around the globe except for Antarctica. The findings revealed that all WBE studies were in accordance with clinical and epidemiological data, which correlates the presence of SARS-CoV-2 ribonucleic acid (RNA) with the number of new daily positive cases officially reported. This last was confirmed via Reverse Transcriptase-quantitative Polymerase Chain Reaction (RT-qPCR) testing which unfortunately is not suitable for real-time surveillance. In addition, WBE concept may act as a faster protocol to alert the public health authorities to take administrative orders (possible re-emerging infections) due to the impracticality of testing all citizens in a short time with limited diagnostic facilities. A comprehensive and integrated review covering all steps starting from sampling to molecular detection of SARS-CoV-2 in wastewater has been made to guide for the development well-defined and reliable protocols.

## 1. Background of Application of Wastewater Based Epidemiology

All the community’s physical, chemical, and biological substances are excreted to the sewer systems and transported to wastewater treatment plants (WWTPs) providing a pooled sample from a group of people in a specific geographical location at a point in time. Risks of emerging infectious diseases and increasing rates of antimicrobial resistance emphasize that infectious disease surveillance is still a fundamental piece of public health. There are some techniques to monitor spatial and temporal trends of diseases such as sentinel surveillance, surveys, mortality and morbidity rates, hospital admission data, human biomonitoring, and wastewater-based methodology (WBE) [1]. This last technique uses “water fingerprinting” to provide an objective and comprehensive assessment of both public and environmental health status in near real-time from water sources (surface waters and domestic wastewaters). Christian Daughton is considered the pioneer of WBE concept [2,3]. He postulated that the evaluation of drug residues (concentration) could be associated with population usage. In 2017, Andrés-Costa et al. estimated that WBE would be a promising tool to collect data on entire communities’ health (or at least in an important percentage) [4]. In this sense, the population’s health condition may be assessed by monitoring biomarkers that included endogenous and exogenous human metabolites as well as different substances. These biomarkers are identified and quantified in untreated wastewaters, and the samples are usually collected in influents of WWTPs that serve communities [5].

In recent decades, WBE, as a novel biomonitoring tool, has been successfully used to investigate polio circulation within the community, monitor the success of international poliovirus vaccine campaigns, investigate the use of some illicit drugs, and provide early warnings of hepatitis A virus and norovirus outbreaks [5,6,7]. Viruses are the main causative agent of several mortal illnesses such as gastroenteritis, hepatitis, and respiratory diseases. After the appearance of Severe Acute Respiratory Syndrome Coronavirus 1 (SARS-CoV-1) in 2003 and Middle East Respiratory Syndrome (MERS) in 2012, the environmental circulation of viruses has received more attention to detect/track human pathogen spread into communities. The importance of surveillance systems became more highlighted with the emergence of coronavirus respiratory disease (COVID-19) in December 2019 in Wuhan, China [1,8]. Since many Severe Acute Respiratory Syndrome Coronavirus 2 (SARS-CoV-2) patients might exhibit few or non-specific symptoms, rapid and accurate diagnosis of potential virus carriers is a critical step to suppress the risk of disease transmission at an early stage [9]. With the first report of SARS-CoV-2 detection in both symptomatic and asymptomatic patient’s feces, several studies have been performed to assess WBE as an early indication tool for COVID-19 transmission and pandemic monitoring [10,11]. Researchers have achieved the identification of SARS-CoV-2 ribonucleic acid (RNA) in different wastewater samples in Netherlands, Australia, France, Brazil, New Zealand, the USA, Japan, and Canada [7,12]. For instance, presence of SARS-CoV-2 RNA has been confirmed in hospital and municipal wastewaters [13,14,15]. Therefore, the World Health Organization (WHO) guideline has been stated for the good management of wastewater [16].

Various studies show a positive correlation between COVID-19 cases and SARS-CoV-2 RNA identification in wastewater. This last confirms that WBE approach is a capable method for early detection, monitoring trends, evaluating the efficiency of public health measures, and tracking immunity of both infected and vaccinated people in response to COVID-19 [7,17]. With the increasing frequency of zoonotic epidemics, WBE can supply future action plans against such pandemics as an environmental surveillance approach [7,18]. As of 2019, only seven studies had investigated the coronaviruses’ presence in environmental compartments such as water, wastewater, and sludge. However, after the emergence of COVID-19, a significant number of studies were conducted over a year, highlighting the importance of WBE studies [7,18]. Although promising, continuous monitoring studies based on different methods are underway, they may provide conflicting results, which indicate that a critical search is necessary to establish a standard virus concentration and identification method for enveloped viruses such as SARS-CoV-2 in water matrix samples. This latter will enhance the accuracy of such surveillance approaches [19,20,21,22].

The present review discusses the background, state-of-the-art, actual status, and prospects of WBE. In addition, detection and quantification methods of SARS-CoV-2 in wastewater covering sampling, storage, inactivation, concentration, extraction and molecular assays are assessed to make a comprehensive and comparable list of studies.

## 2. Cutting-Edge of Wastewater Based Epidemiology Concept

WBE is based on the extraction, detection, analysis, and interpretation of chemical/biological compounds (biomarkers). Subsequently, this methodology gives information about health community and environmental exposure, as mentioned above. Genetic biomarkers are crucial for determining the disease incidence. A suitable genetic biomarker must have the following characteristics: stable (in sampling and storage), specific for a particular disease, consistent between distinct genders and ethnic groups, human-specific and excreted in urine or feces constantly, and not absorbable to particulate matter, for instance, deoxyribonucleic acid (DNA), RNA, or antibiotic resistance genes [23].

The main advantages of WBE method are: (i) asymptomatic and pre-symptomatic patients may be detected (keeping the anonymity of individuals); (ii) evidence of SARS-CoV-2 circulation to support public health measures and limit the transmission; (iii) helps to identify hotspots for further classical surveillance interventions; and (iv) a non-invasive, viable, and almost real-time method. However, the main disadvantage is the matrix complexity (wastewater) together with loss of biomarkers within the drain system and during storage. Another important consideration to keep in mind is the dynamic population (tourism/business activities) [24] and the weather variations (geographic location). Although WBE has many advantages, it should not be viewed as a replacement for clinical testing. Still, it can provide independent information to public health decisions.

SARS-CoV-2 DNA/RNA residues present in raw wastewater indicate that COVID-19 disease is circulating inside the community. Unfortunately, the lengthy incubation time and virus shedding have allowed the spreading of SARS-CoV-2 causing a poor containment. The knowledge of the occurrence of SARS-CoV-2 in wastewater and secondary sludge from WWTPs could predict the decreasing or rising trends (second or more infectious waves). Kumar et al. concluded that high viral concentration goes hand in hand with the high number of COVID-19 infected individuals [25]. Similar studies have reported the occurrence of SARS-CoV-2 in fecal and urine samples [26,27,28,29,30,31,32]. It is important to note that wastewater generally contains organic matter, particulate solids, micro and macro pollutants, and other pathogens. Moreover, SARS-CoV-2 contained in feces usually undergoes several transformations along the sewer network, and consequently, the dilution factor must be considered. Wastewater properties and sewer conditions cause a reasonable uncertainty (20–40%) [33]. In the same vein, disinfection technologies (thermal, chlorine species, ozonation, and UV radiation, among others) usually inactivate the SARS-CoV-2 virus due to the similarities in phylogenetic with SARS-CoV-1. Without active human cells as hosts in wastewater, the infectivity of SARS-CoV-2 could be reduced up to 90% in minutes. However, SARS-CoV-2 RNA was significantly more persistent (3–33 days) [33]. The resulting non-infectious virus could still be detected by Reverse Transcriptase Polymerase Chain Reaction (RT-PCR) based methods [34]. Zhang et al. investigated the presence of SARS-CoV-2 RNA by Reverse Transcriptase-quantitative Polymerase Chain Reaction (RT-qPCR) in influent and effluent of septic tanks located at a hospital in China. Although the occurrence of SARS-CoV-2 viral RNA was not found in the influent sample, the effluent of septic tank tested positive for such viral RNA, even after the second stage of disinfection [35]. This last is attributed to the shield provided by suspended solids by embedding viruses during disinfection. In conclusion, disinfection step may lead to viral loss; however, health and well-being of the operating/laboratory personnel is a priority. Moreover, studies to refine WBE procedures and, consequently, to improve estimates are urged.

According to the Commission Recommendation (EU) 2021/472 on a common approach to establish a systematic surveillance of SARS-CoV-2 and its variants in wastewaters in the EU [36], WBE surveillance should be taken into account as a complementary and independent tool to COVID-19 surveillance and testing strategies. Therefore, WBE requires to be included more systematically in the national testing strategies for tracing of the SARS-CoV-2 virus. In addition, the Member States are strongly encouraged to put in place as soon as possible and no later than 1 October 2021 a national wastewater surveillance system targeted at data collection of SARS-CoV-2 and its variants in wastewaters.

Finally, WBE serves to observe trends on SARS-CoV-2 spreading and could be an effective early warning for possible COVID-19 re-emerging infections. Moreover, local health authorities can use this knowledge to take administrative orders related to notify the potential health threat and diminish the possibility of transmission. Updated data have confirmed that surveillance of SARS-CoV-2 and its variants in wastewaters can supply a cost effective, rapid, and reliable source of information.

## 3. Actual Status of Wastewater Contamination with SARS-CoV-2

The COVID-19 wastewater-based epidemiology, other than the droplet or aerosol-based transmission, needs to be emphasized [37,38,39,40]. The SARS-CoV-2 genome load has been predicted to be 600,000 per mL of feces [41], and it is known that one COVID-19 patient can produce up to 370 L water per day [42]. The SARS-CoV-2 virus can remain intact in various media [43,44,45]. Therefore, the virus can be transported to water bodies. The importance of epidemiology based on different sources of wastewater together with its environmental implication has had a greater emphasis nowadays [46,47,48,49]. The change of position of the virus caused by water flows can produce a quick evolution of the same, as observed in the case of the subsequent waves of the COVID-19 disease in many countries [50,51]. It is clearly known that an infected person who sneezes, coughs, talks or sings can infect a healthy person through droplets of various sizes. Similarly, if COVID-19 patients use the sink, bathroom, toilet or basin, the possibility of transmitting the virus to the environment is logically accepted and studied [48,49,52,53].

**Figure 1:**
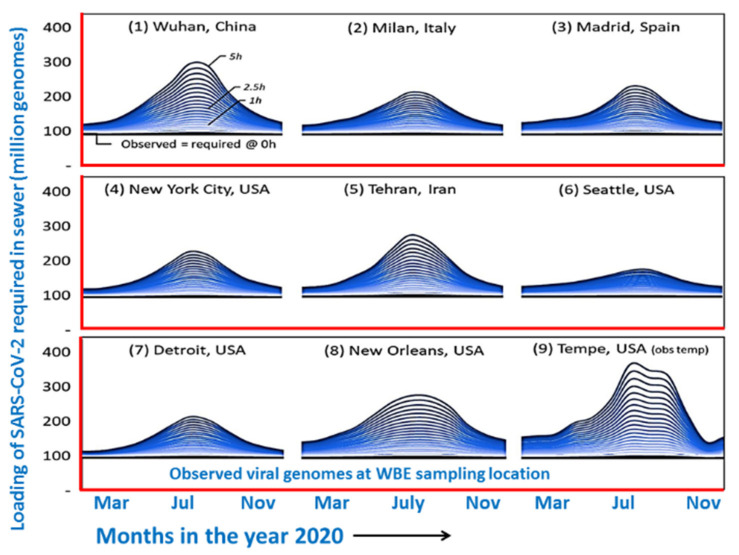
Predicted water borne viral load in different cities around the world (Redrawn after Hart et al. [42] under CC BY-NC-ND 4.0 license).

Environmental surveillance for both the self-quarantined or isolated patients and from COVID-19 hospitals to identify and control the spreading of COVID-19 has been emphasized very early [41,48,54]. Worldwide reports, as observed below, have confirmed that the viral RNA exists in several wastewater samples. This last could make the situation worse for the disease’s outbreak. The RNA of SARS-CoV-2 was argued to come from waste such as stools, urine, washing materials, clothes, etc. of COVID-19 patients [52,53,54]. As per the infection rate observed in different cities around the globe (Figure 1), the viral loading about 100–300 million genomes in drainage would happen before the wastewater treatment from COVID-19 care centers and isolation homes. Data were well correlated as per the cases noticed in those cities.

Still now, the theory of zoonotic origin of the virus has been established. However, the urination, defecation, and sneezing activities of infected patients or asymptomatic persons cause the incidence of SARS-CoV-2 RNA in drainage systems. Then, healthy people may be infected via drinking water or other activities using contaminated water.

As vaccination campaigns increase, the viral load of SARS-CoV-2 in wastewater samples hardly relates to the reported cases due to the new asymptomatic cases [55]. However, medium or low SARS-CoV-2 RNA concentrations in wastewater may prevent possible new waves, and vaccines may be directed by governments to specific areas [56,57]. For instance, La Rosa et al. [58] confirmed the wastewater contamination by SARS-CoV-2 RNA two months before the disease outbreak in Milan and Turin. A similar report was also documented by Chavarria-Miro et al. [59] in Barcelona 41 days before the onset of the disease in 2020 [59]. In Bnei municipality area of Israel, a positive correlation was observed between the RT-PCR test and the wastewater samples of infected cases in March 2020 [60]. Wurtzer et al. [61] also observed a similar association between SARS-CoV-2 RNA detection in wastewater and the number of confirmed cases with an 8 day temporal shift in Paris. Interestingly, a similar exponential viral load was detected in municipal wastewater in Massachusetts from early January to May 2020 before the confirmed cases were documented [62].

**Figure 2:**
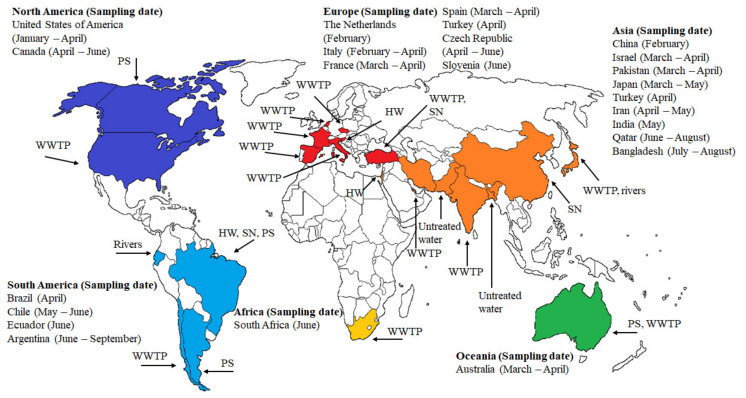
Overview of the first detection of SARS-CoV-2 in wastewater around the world from January 2020 to February 2021. Sampling point: wastewater treatment plant (WWTP), suburban pumping station (PS), hospital wastewater (HW), and sewer network (SN).

Several analyses have been performed to detect the presence of SARS-CoV-2 in all continents (except Antarctica). As shown in Figure 2, the first detection of SARS-CoV-2 in different types of waters such as WWTPs, suburban pumping stations (PS), hospital wastewater (HW), and sewer networks (SN) around the globe are reported in Figure 2. All sampling dates were in 2020. Presence of SARS-CoV-2 RNA in wastewater was first reported in a WWTP at USA (Louisiana) from 13 January to 8 April. About 28.57% (2/7) of samples were reported positives [63]. In Canada (Ottawa and Gatineau), RT-qPCR analyses showed an increase in the rate of documentation of N1 and N2 genes primary in sludge (92.7, 90.6%) as compared to influent post grit solids samples (79.2, 82.3%). The authors mentioned that after statistical treatment of the data, a strong positive association was noticed between the copy number of the viral RNA and number of confirmed cases from April 2020 to June 2020 [64]. A similar report during inlet and outlet of preprocessing and disinfection in the sewage network from 19 to 24 February was also documented in China (Zhejiang). The samples showed 100% detection rate of SARS-CoV-2 RNA in the sewage water [15].

The Netherlands (Haarlemmermeer) was the first European country where the SARS-CoV-2 viral RNA (RT-qPCR test) was detected in untreated wastewaters (from 17 to 24 February) [17]. Then, about 50% positive tests (6/12 samples) for the virus in WWTP were detected at Milan and Rome, Italy, from 3 February to 2 April [65]. Spain (Murcia) and France (Paris) analyzed samples in WWTPs from March to April. In Spain, the sampling points were the untreated wastewater, and the effluent after the secondary and tertiary processes. Viral RNA detections in influent, secondary effluent, and tertiary effluent were 83.33% (35/42 samples), 11.11% (2/18 samples), 0.0% (0/12 samples), respectively. The data revealed that community shedding of SARS-CoV-2 RNA via fecal discharge was occurring in many cities before the wastewaters were sampled for testing [14]. In a WWTP located in Paris, viral RNA detection was 100.0% (3/3 samples) [61]. Samples in WWTPs form Turkey (Istanbul, from 21 to 25 April) and Czech Republic (from April to June) indicated a 71.42% (5/7 samples) and 27.3% (9/33 samples) of the viral RNA detection, respectively [66,67].

Adding to the count, hospital wastewaters samples from Slovenia (Ljubljana, 1 to 15 June), Brazil (Rio de Janeiro, April), and Israel (Tel Aviv, 10 March to 21 April) were positive detected as 66.7% (10/15 samples), 41.6% (5/12 samples), and 38.4%, respectively [68,69,70]. The reports were good correlated with the high COVID-19 patients cases reported during the mentioned periods. Surprisingly, studies from Japan (Yamanashi Prefecture, from 17 March to 7 May) and Ecuador (Quito, June) revealed that the SARS-CoV-2 RNA was present in natural waters (rivers). Adding to the fact in Japan, the viral RNA detection was 20% (1/5 samples) when the COVID-19 cases peaked in the community [71], whereas in Ecuador, the viral RNA detection was documented in all the three samples [72]. Studies in South America (Chile and Argentina) reported the occurrence of the virus in untreated wastewater and suburban pumping stations. In Chile (Santiago, from March to June), the viral RNA was noticed in the influent and effluent sampling points. In Argentina (Buenos Aires, from 5 June to 7 September), all 11 samples collected from wastewater showed a viral load [73]. In this case, it is important to highlight that SARS-CoV-2 RNA was not detected during the first two months of analyses. However, in May and June, SARS-CoV-2 viral load was progressively increased in wastewater bodies [74].

The occurrence of viral SARS-CoV-2 RNA has been analyzed in the Gomoti river of India [75]. In Asia, India (Ahmedabad, from 8 to 27 May), Qatar (Doha, from 21 June to 30 August), and Iran (Tehran, Qom, and Anzali, from 4 April to 2 May), the presence of the virus in WWTPs was noticed. Viral RNA detection analyses showed 100% (2/2 samples), 100% (43/43 samples), and 66.66% (4/6 samples) in influent samples collected from the above countries, respectively [25,76,77]. The trend of the detection of SARS-CoV-2 RNA analyzed by RT-qPCR testing in wastewater in these countries was matched by the number of new daily COVID-19 positive cases. Bangladesh (Noakhali, from 10 July to 29 August) was entered into the club when viral RNA (75.0% (12/16 samples)) in sewage waste tanks, passage drains, and toilets was detected [13]. Finally, in Pakistan (Islamabad, from March to April), the viral RNA detection was 27% (21/78 samples) in the sewage network [78]. Therefore, almost all South Asian countries have well documented the SARS-CoV-2 viral load in wastewater.

Lastly, in Oceania (Queensland, Australia, from 27 March to 1 April), through Monte Carlo simulation, the number of COVID-19 cases estimated in the catchment agreed with clinical observations. Viral RNA detection was reported as 22.2% (2/9 samples) [79]; and in Africa (Western Cape, South Africa, June), the Viral RNA detection was found to be 100.0% (5/5 samples) in WWTPs. The results showed that the presence of SARS-CoV-2 data has corresponded with the COVID-19 cases [80].

By way of conclusion, the detection of SARS-CoV-2 RNA in wastewater indicates a strong association of the COVID-19 disease via WBE [81]. Proper hygienic knowledge and implications of COVID-19 guidelines are put forth by many world-class health organizations including the Centers for Disease Control and Prevention of US (CDC-US), WHO, etc. Just as the use of masks is mandatory to restrict the mode of infection transmitted by air, the treatment of wastewater before discharge into the tanks is also equally important. Strong policies and awareness should be adopted for the treatment of wastewater matrices of COVID-19 patients as well as to their corpses and materials for daily use to control such disease.

## 4. Molecular Detection of SARS-CoV-2 in Wastewater and Treatment Plant Sludge

Until today, many WBE surveillance studies have confirmed the detection of SARS-CoV-2 genetic material in wastewater and solids/sludge originated from WWTPs located in cities all over the world. In these studies, the followed protocols mainly encompassed the following steps: (i) the selection of an urbanized area drained by the sewer system, (ii) wastewater samples collection, (iii) biomarker selection (e.g., RNA, anti-inflammatories, IgM/IgG), (iv) sample pretreatment and instrumental analysis, (v) quantifying of SARS-CoV-2 RNA copies in wastewater (concentration methods) and data analysis, (vi) comparison between WBE and other data sources (validation), (vii) comparison of WBE results with other studies (other countries), and (viii) analysis of uncertainties and limitations. Some steps are introduced and discussed in detail in the following subsections.

### 4.1. Sampling

Wastewater samples were collected from different sites in WWTPs or manholes (MH). Some research groups performed their SARS-CoV-2 detection and quantification studies using composite samples, which were prepared by combining portions of multiple grab samples or using automatic sampling devices. Others preferred to use grab samples, as indicated in Table 1 and Table 2. The solid and sludge samples associated with wastewater treatment were taken as grab samples representing a snapshot in all studies.

### 4.2. Storage

Storage and shipment of the sample are of great importance in terms of virus survival. Most samples were shipped on ice or with cold packs [101]. Some samples were stored in a refrigerator at 4 °C, while others were frozen in a freezer at a ranged temperature between −20 °C and −80 °C, after the concentration step, for further processing and analysis, as shown in Table 1 and Table 2. As of now, few studies have investigated the effect of the storage temperature on the SARS-CoV-2 RNA copies in wastewater [76,77]. The persistence of SARS-CoV-2 genetic material at 4, −20, and −75 °C was assessed using RT-qPCR assays targeting E-Sarbeco and N2 for a time interval of 29, 64, and 84 days [77]. Findings confirmed the stability of the SARS-CoV-2 RNA in cold storage, particularly at frozen conditions. Data of Baldovin et al. [76] also confirmed the presence of SARS-CoV-2 RNA in the refrigerated samples for a 24 h storage period.

### 4.3. Inactivation of SARS-CoV-2

A heating protocol, screening and elevating temperature from 56 to 92 °C, was performed using clinical samples collected from COVID-19 patients. This protocol was used to ensure laboratory personnel and the environment’s safety [102]. A lower inactivation performance (5 log10 reduction) was exhibited at 60 °C for 60 min. However, a drastic reduction in RNA copies detection was determined at 92 °C for 15 min, which yielded a total inactivation of SARS-CoV-2 virus. Heating protocol allowed the detection and quantification of RNA copies in an acceptable interval. Based on results, the author has recommended the inactivation protocols at 56 °C for 30 min and 60 °C for 60 min for SARS-CoV-2 virus [103]. A few studies initiated the inactivation of SARS-CoV-2 virus at 60 °C for 90 min [60,64,67].

### 4.4. Preconditioning

Most wastewater samples, before the concentration step to remove bacterial debris and large particles, use centrifugation and/or filtration methods. As shown in Table 1 and Table 2, the samples have been centrifuged at a varied speed from 1200× *g* to 5000× *g* for a time interval from 5 min to 45 min. Only one research performed the centrifugation method at an extreme speed of 24,000× *g* at 4 °C for 30 min [104]. Some molecular assays performed filtration process using a 0.22 μm pore size filter. Generally, the partition of SARS-CoV-2 virus to the solids in wastewater was ignored, except for some studies [49,85,99]. Kocamemi et al. [99] shook the sludge samples produced after the wastewater treatment. These samples were stirred at 100 rpm and 4 °C for 30 min to transfer SARS-CoV-2 viruses into the liquid phase before the pre-centrifugation at 7471× *g* for 30 min. Kitamura et al. [85] and Westhaus et al. [49] analyzed the solids obtained from the pre-centrifugation step separately. A loss of solids has already been mainly reported for enveloped viruses [105].

### 4.5. Concentration

**Figure 3:**
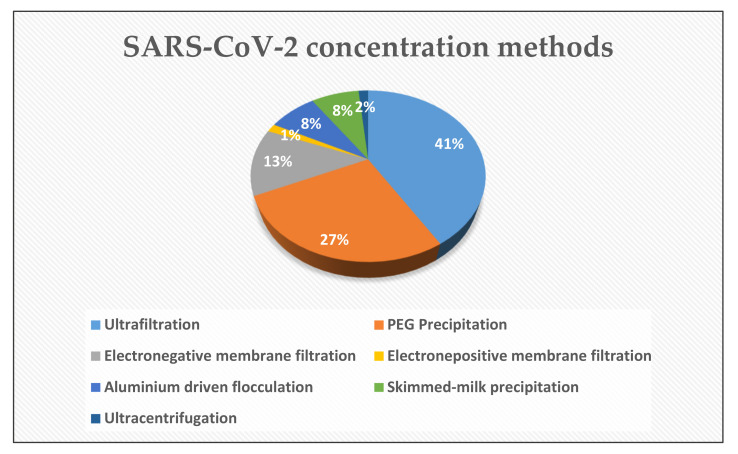
Pie chart showing the SARS-CoV-2 concentration methods used within the context of the WBE surveillance.

Since the viral load is severely dilute in a huge volume of wastewater, the concentration method utilized for the recovery of SARS-CoV-2 genetic material plays an integral role to maximize the overall performance of molecular assays. Until now, several concentration methods such as (i) ultrafiltration (UF), (ii) precipitation with polyethylene glycol (PEG), (iii) electronegative or electropositive membrane filtration, (iv) the aluminum-driven flocculation, and (v) the skimmed-milk flocculation (SMF) have been applied. The Figure 3 shows the percentage distribution of mentioned techniques. It is worth emphasizing that all of them have been well established and documented for the non-enveloped viruses such as polioviruses, noroviruses, and adenoviruses [105,106,107]. Within the context of the WBE surveillance, UF and PEG are the most utilized techniques for the concentration of SARS-CoV-2 genetic material from wastewater [17,25,49,63,76,77,79,83,84,85,86,87,88,90,91,93,95,99,104,108]. These studies are compiled from the recent literature and presented in Table 1 and Table 2.

SARS-CoV-2 genetic material has been commonly concentrated from wastewater via centrifugal filters with a nominal molecular weight limits (NMWL) ranging from 10 kDa [26,66,76,77,83,84,89,90], 30 kDa [88,93,97,98,101], 50 kDa [109], and 100 kDa [23,92] to 150 kDa [87,93] at centrifugation speeds varying in the range from 3000× *g*–4000× *g*. The protocols followed by these studies are summarized in Table 2. Additional to the centrifugal ultrafiltration (CeUF), hollow fiber ultrafilters (HFUF) composed of polysulfone PVP high flow pipettes were also tested [93,95]. Data obtained from CeUF and HFUF proved that UF is one of the most applicable and sensitive promising concentration techniques for the recovery of SARS-CoV-2 genetic material from wastewater and wastewater treatment plant sludge. However, the sensitivity of UF could be improved by its double application [84]. The application of either CeUF or PEG precipitation to HFUF concentrated is an unsuccessful and unnecessary secondary concentration method, according to Gerrity et al. [95].

The PEG precipitation method was firstly proposed by Albertsson and Frick [110]. This technique is based on the partition of some proteins in a liquid two-phase system, composed of dextran, methylcellulose, and water. This method has been tailored, well-documented, and successfully applied for the concentration of enteroviruses in groundwater, river water, tap water, and wastewater since 1960s [111,112,113]. WHO has also recommended a modified version incorporating dextran addition of PEG precipitation for poliovirus circulation’s environmental surveillance [114]. Due to its well-known success in the detection of poliovirus, many researchers have applied PEG precipitation with slightly different implementations or modifications to the composite, grab wastewater, or sewage sludge samples collected from different sources (manholes and WWTPs) located in the Republic of Turkey [66,99], India [25,75], Italy [58], USA [62,115], Argentina [73], Japan [97], Spain [14,59,90,93], Israel [60], Canada [64], the United Arab Emirates [86], and France [87] to recover SARS-CoV-2 genetic material as indicated in Table 2. Moreover, it has been successfully detected and quantified by its different implication like overnight standing [25,71,87,91,97,116]. Positive results were also achieved by the PEG-Dextran method [114] and its modification [100,117,118]. Some research groups employed another modified version of PEG precipitation proposed by Wu et al. [62,85,87,115,118]. However, this modified version yielded inconsistent data but lower inconsistency than other concentration techniques such electronegative membrane filtration (ENMF) [85] or UF [85,86].

Virus adsorption–elution (VIRADEL) utilizes electrostatically charged microporous materials as filtration media [105,111]. Based on their surface charges, VIRADEL can be classified as electropositive membrane filtration (EPMF) and ENMF. The RNA recovery efficiency of ENMF can be enhanced by adding MgCl_2_ or NaCl to support the attachment of virus particles onto a cellulose nitrate membrane filter with a pore size of 0.45 µm via salt-bridging. VIRADEL has been adopted as a standard method “US EPA Method 1623: Cryptosporidium and Giardia in Water by Filtration/IMS/FA” by US EPA [118]. In few studies, ENMF with/without support with MgCl_2_ was used and suggested as a suitable concentration technique for the WBE SARS-CoV-2 surveillance [13,71,83,119]. Only one study performed this technique to concentrate SARS-CoV-2 RNA from wastewater using an electropositive NanoCeram column filter [119].

Based on the viruses’ elution with glycine alkaline buffer prior to organic flocculation with skimmed-milk flocculent, SMF, is another concentration technique tested within the context of SARS-CoV-2 WBE surveillance. This technique exhibited superior performance to concentrate SARS-CoV-2 genetic material from wastewater [72,120]. Finally, aluminum-driven flocculation based on the capability of freshly formed Al(OH)_3_ in the adsorption of the viruses provided an efficient SARS-CoV-2 RNA recovery [14,96].

### 4.6. Extraction

Isolation of SARS-CoV-2 RNA from the concentrated sample without damage is another important step that may drastically affect the overall detection and quantification performance. Several techniques based on extraction with organic solvents, silica membrane-based spin column, and the use of paramagnetic particles [121,122] have been adopted and refined for this purpose. As seen in Table 1 and Table 2, all extraction approaches use acid guanidinium thiocyanate–phenol–chloroform (TRIzol-chloroform), commercial kits based on solvent extraction utilizing TRIzol-chloroform, lysis buffer/TRIzol LS, or silica membrane-based spin column except the paramagnetic particle’s method. The CDC-US has qualified and validated several commercial RNA extraction kits for SARS-CoV-2 which are mentioned on the webpage of CDC-US [123].

### 4.7. Detection and Quantification

Quantification of SARS-CoV-2 genetic material is the last and the utmost important step of a molecular assay. RT-PCR and RT-qPCR have been globally accepted as standard methods for quantifying RNA viruses [13,62,124,125,126]. These molecular tests offer high sensitivity and specificity. However, these analyses require quite complex sample handling in the laboratory, expertise personnel, and a long time of data processing and analysis (4–6 h). Likewise, Clustered Regularly Interspaced Short Palindromic Repeats (CRISPR) and digital PCR (dPCR) have been evaluated [26]. Findings indicated that the reverse transcription droplet digital PCR (RT-ddPCR) is also an alternative technique for the detection and quantification the SARS-CoV-2 RNA in wastewater. The research groups have generally used RT-PCR and/or RT-qPCR (Table 1 and Table 2). A few research groups have carried out the molecular assays using RT-ddPCR [64,87,104,127]. Some of them also compared the performance of R-ddPCR with RT-qPCR [66,87,95]. Different sets of primers/probes targeting different parts of viral particles were used to amplify SARS-CoV-2 RNA extracted from wastewater/sludge samples. For instance, 2019-nCoV_N1(-F; -R; -P), 2019-nCoV_N2(-F; -R; -P), 2019-nCoV_N3(-F; -R; -P), E_Sarbeco(_F; _R; _P1), Cor-p-F2(+), and Cor-p-R1 (-) were used to target nucleocapsid (N) and envelope (E). In some studies, RdRp (ORF1a and ORF1b) and S genes were also targeted. A list of “Only 2019-Novel Coronavirus (2019-nCoV) Real-time RT-PCR Primers and Probes” has already been published by CDC-US (CDC, 2019) for respiratory virus surveillance and research purposes.

To validate the SARS-CoV-2 detection and quantification, molecular assays have been performed using domestic wastewater samples [14,49,91,100,128,129] as well as clinical samples collected from the COVID-19 patients [130,131,132]. Studies focused on designing these modern molecular techniques have been already continued [116,133,134] to develop a gold standard for the detection and qualification of SARS-CoV-2.

Additionally, the aforementioned molecular assays do not provide information on viability of SARS-CoV-2 being present in the water matrix [77]. To gather information on the virus viability, specific molecular techniques such as ethidium monoazide (EMA)-RT-qPCR, propidium monoazide (PMA)- RT-qPCR, or integrated cell culture-RT-qPCR, could be established.

In summary, modern molecular techniques produce accurate data when validation and standardization studies are completed. However, these techniques are not suitable for real-time surveillance due to their skilled operator requirement and expensive initial costs. Therefore, the development of easy-to-operate, cost-effectively, and fast response equipment for online monitoring is another urgent issue to be searched for the WBE SARS-CoV-2 surveillance.

## 5. Conclusions

The conclusions and future directions of the present review can be drawn as follows:

- Recent data proved that SARS-CoV-2 RNA can be detected in human feces from a few days to a week before the onset of symptoms. Therefore, the monitoring of SARS-CoV-2 genetic signals in wastewater samples seems to be a useful methodology for prediction of COVID-19 outbreaks. However, at the early stage of COVID-19 prevalence, its surveillance in sewage network may not be quite accurate due to the extremely low concentration of virus in wastewater.
- One liter of wastewater is like an ocean of information that can provide valuable data about the timing and prevalence of COVID-19 outbreaks in communities, especially in low-income countries with low clinical COVID-19 testing rates.
- Periodically collecting wastewater samples from sewer networks for the trace of the SARS-CoV-2 virus can be effective as a primary non-clinical warning tool for early detection.
- Considering that SARS-CoV-2 RNA has already been detected in the activated sludge, the dissociation of SARS-CoV-2 genetic material from solids/semisolids in wastewater arises urgent issue to be sought for accurate quantification. In this sense, a protocol tailored to SARS-CoV-2 is another crucial need.
- Although the sampling method is one of the most important steps of WBE SARS-CoV-2 surveillance, there is no real consensus on adequate and representative wastewater sampling technique. Then, further studies are required to find out a properly designed and well-defined sampling procedure.
- Among the concentration techniques, CeUF, double UF, and PEG precipitation seem to be promising concentration techniques for SARS-CoV-2 RNA enrichment from wastewater/sludge after re-partitioning.
- Findings prevailed that more standardization and quality control studies covering (i) selection of appropriate sample volume, (ii) improvement of preconditioning step including the significant contribution of the solid fraction of raw wastewater, (iii) spike controls, (iv) PCR inhibitor removal, and (v) selection of suitable extraction approach must be performed to maximize the SARS-CoV-2 recovery efficiency.
- An urgent issue to be faced is the development of molecular techniques providing information on the viability of the virus in the sewage system for worker safety.
- Recent data have revealed a correlation between genetic material in wastewater and the number of clinically reported positive cases confirming that WBE surveillance could be utilized as a sensitive tool to trace the circulation of SARS-CoV-2 virus in the population. Nevertheless, an accurate mathematical model considering several factors such as wastewater characteristics, climate, and sewage system structure, among others, should be developed.
- WBE for SARS-CoV-2 is a fast and effective surveillance system with the clearest potential to avoid and control the infectious disease outbreak.

## Figures and Tables

**Table 1:** The methods used for SARS-CoV-2 detection in wastewater and sewage sludge.

Sample	Volume	Preconditioning Step	Concentration/Extraction/Detection	Reference
Single sample from WWTP influent in Brisbane, Australia	100–200 mL	**UF1** Centrifuge the sample at 4500× *g* for 10 min at 4 °C **Ultracentrifugation** Centrifuge at 100,000× *g* for 1 h at 4 °C. **PEG precipitation** Centrifuge at 10,000× *g* for 20 min at 4 °C to remove larger particles and debris. Transfer the supernatant to a new centrifuge tube Store the supernatant at 4° (S1)	**UF1**: Centrifuge at 4750× *g* for 10 min through centrifugal filter (30 kDa) **UF2**: Centrifuge at 3500× *g* for 30 min through centrifugal filter (10 kDa) **ENMF 1**: Filtrate through 0.45 μm of ENM **ENMF 2**: After addition MgCl_2_ (final concentration of 25 mM), filtrate 0.45 μm of ENM **ENMF 3**: After adjusting pH 4.0 (2 N HCl) pass through membrane filter (0.45 μm) **Ultracentrifugation (UFC)**: Re-suspend the pellet in 3.5 mL of 0.25 N glycine buffer (pH 9.5), incubate it on ice (30 min), add 3 mL of 2 × PBS (pH 7.2), centrifuge eat 12,000× *g* for 15 min at 4 °C, UFC at 100,000× *g* for 1 h at 4 °C to recover the virus, re-suspend the pellet in PBS (pH 7.2) **PEG Precipitation**: Re-suspend the pellet in beef extract (3% *w*/*v*) in 0.05 M glycine (pH 9.0) at a ratio of 1:5; agitate at 200 rpm for 30 min at room temp., centrifuge at 10,000× *g* for 10 min at 4 °C, transfer the pellet suspension into S1; neutralize pH (2 M HCl), add PEG 8000 (10%) and NaCl (2% *w v*^−1^), incubate at 120 rpm at 4 °C for 2 h, centrifuge it at 10,000× *g* at 4 °C for 30 min; discard the supernatant; re-suspend the pellet in 800 μL Trizol, further steps: as described in Ahmed et al. [82].	[83]
Composite samples from a PS and two WWTPs in Southeast Queensland, Canada	100–200 mL	**UF** Centrifuge the sample at 4750× *g* for 30 min at 4 °C	**UF**: Centrifuge at 3500× *g* for 15 min through centrifugal filter (10 kDa); invert and place the concentrate cup on top of the sample filter cup, centrifuge at 1000× *g* for 2 min. **RNA Extraction—PCR assay**: RNeasy PowerMicrobiome Kit^®^ via QIAcube Connect platform **RT-qPCR**: Bio-Rad CFX96 thermal cycler, targeting gene: N protein	[82]
Untreated municipal samples from three different regions in Stockholm, Sweden, and one region from the North of Italy	N.A.	As described in the next column	**UF**: Centrifuge at 4600× *g* at 4 °C for 30 min; filter centrifugal ultrafilter (10 kDa) at 1500× *g* for 15 min **Double UF**: Application of UF twice **Adsorption-extraction-ENMF**: Add MgCl_2_ (25 mM), filtrate (0.45 μm-ENMF) **Centrifugation-adsorption-extraction-ENMF**: Centrifuge at 4600× *g* at 4 °C for 30 min, add MgCl_2_ (25 mM) to the sample, filtrate (0.45 μm-ENMF) Extract RNA obtained UF and double UF by TRIzol-chloroform using miRNeasy™ Mini Kit Extract RNA obtain from ENMFs using RNeasy™ Power Microbiome Kit RTqPCR analysis (Primers targeting the nucleocapsid (N) gene)	[84]
Grab WW samples, WWTPs in Italy	100 mL	Filter on 0.22 μm polyether sulfone (PES)	**UF**: Filtrate through centrifugal 10 kDa filter units in a swinging bucket rotor at 4000 × *g* for 10 min, RNA extraction: QIAamp™ viral RNA mini kit; StepOnePlus™ Real-Time PCR System	[17]
24 h composite and grab wastewater samples; WWTPs in Louisiana, USA	250 mL	Centrifuge the sample at 3000× *g* for 30 min	**UF**: Centrifuge at 1500× *g* for 15 min through centrifugal filter (100 kDa); invert and centrifuge the filtrate unit at 1000× *g* for 2 RNA extraction using ZR™ Viral RNA Kit (RT-qPCR) assays (CDC N1 and N2)	[63]
Grab; 2 WWTPs; MH in Japan	400 mL	Centrifuge at 3000 rpm (1840× *g*) for 30 min	**UF**: Following instructions of Ahmed et al. [82] using centrifugal filter (30 kDa), further steps as described in Table 2	[85]
24 h composite, 11 WWTPs, MH, PS		Pasteurize in a 60 °C water bath for 90 min Filter through 0.22 μm polyethersulfone filter	**UF**: Centrifuge polypropylene (PP) concentration/spin column (30 kDa) Further steps as described in Table 2	[86]
Grab samples (after the decantation before the activated sludge process) in French Grand Est	2 volumes of 50 mL	Not applied	**UF**: Centrifuge at 1500× *g* for 15 min a centrifugal ultrafilter (100 kDa), inverting the system and applying centrifugation (1000× *g* for 2 min), wash the ultrafilter with 3.5 mL deionised water, combine washing water (3.5 mL) with the concentrate (1.5 mL), apply further two washing steps using 5 mL NucliSENS^®^ lysis buffer (bioM’erieux) for an incubation time of 5 min, use the entire for nucleic acid extraction	[87]
Grab and composite samples in Japan	40 mL	Centrifuge at 3500× *g* for 5 min	**UF**: Centrifuge at 3500× *g* for 20 min a centrifugal ultrafilter (30 kDa) Further steps as described in Table 2	[88]
24 h composite samples; WWTP in Montpellier, France	50 mL (COS)	Centrifuge at 4500× *g* for 30 min at 4 °C Pass the supernatant through a 40 μm cell strainer Froze at −20 °C for further analyses	**UF**: Centrifuge through centrifugal ultrafilter (50 kDa) **RNA extraction**: the NucleoSpin™ RNA Virus kit TaqPath One-Step RT-qPCR, CG master mix	[85]
^A^ Mixture comprised of 3 grab samples	50 mL	Centrifuge at 4500× *g* for 30 min Filtrate through 0.22 μm membrane filters	**UF**: Concentrate (30 times) the filtrate using the 96 well filter plate (10 kDa) Further steps as described in Table 2	[89]
^B^ 24 h composite WW samples		Centrifuge at 4500× *g* for 30min	**UF**: Centrifuge through centrifugal ultrafilter (10 kDa), buffer pH to 7.4 with PBS Further steps as described in Table 2	[90]
^C^ Samples from in İstanbul, Turkey	250 mL	Centrifuge at 3200× *g* for 45 min	**UF**: Centrifugation at 3200× *g* for 25–40 min, through centrifugal ultrafilter (10 kDa), Further steps as described in Table 2	[66]
24 h composite WWTP in Southeast England	120–240 mL	Centrifugation at 3200× *g* Filter through 0.45 μm filter	**UF**: Centrifuge through centrifugal filter (10 kDa) Real-time RT-qPCR using a qScript XLT qPCR Toughmix system	[91]
Samples from 3 WWTPs in Zurich (A), Lausanne (B), in a resort Switzerland	50 mL	On the sample taken from a WWTP Filtrate through 2 µm and 0.22 µm filters On the sample taken from WWTPs B Centrifuge at 4863× *g* for 30 min	**UF**: Concentrate the supernatant using centrifugal filter units (Centricon^®^ Plus-70 Ultrafilter, 10 kDa) by centrifugation at 3000× *g* for 30 min RNA extraction by the QiaAmp™ Viral RNA MiniKit, store the extracted sample at −80 °C cDNA transcripts using NGS; Sequencing using the Illumina NovaSeq 6000 platform	[92]
24 h-composite raw wastewater samples from 6 WWTPs, in Catalonia, Spain	200 mL	Seed an aliquot of 200 mL of the sample with 107 GC mL^−1^ of MS2 and MHV (1:100, *v v*^−1^) Centrifuge at 4750× *g* for 30 min	**UF1**: Concentration Pipette CP-Select™ using Hollow Fiber Polysulfone PVP high-flow pipette (150 kDa), elute viral particles with 0.075% Tween-20/Tris using Wet Foam Elution™ cans **UF2**: Centrifuge through centrifugal ultrafilter (30 kDa) at 3000× *g* for 30 min Elute the virus by inverting the CeUF device, centrifuge at 1000× *g* for 3 min **RNA extraction**: the QIAmp Viral RNA Mini kit	[93]
^D^ Water/wastewater composite and grabs samples	150 mL	Filter only samples from one facility with 100-mm filter paper and others as is	**HFUF**: Hollow fiber ultrafiltration (30 kDa) **UF**: Centrifugal ultrafiltration (30 kDa or 100 kDa) Centrifuge at 3500× *g* for 15–30 min at 10 °C, analyse as-is or further processed with 2nd concentration via Centricon ultrafilters or PEG precipitation according to [94] **Extract RNA and DNA** using Purelink Viral™ RNA/DNA Mini Kit CFX96 or CFX384 Touch™ Real-Time PCR Detection Systems	[95]
24 h composite WWTP, in Helsinki, Finland	60 mL	Centrifuge at 4654× *g* for 30 min	**UF**: Centrifuge through centrifugal filters (**10 K**) at 3000× *g* for 25 min, followed by concentrate collection with 1000× *g* for 2 min (~400 μL of concentrate)	[77]
24 h flow-dependent composite influent and effluent samples, 6 WWTPs in Germany		**Liquid phase**: Centrifuge at 4700× *g* for 30 min **Solid phase**: Centrifuge at 4700× *g* for 30 min Wash the separated pellets with deionized water Centrifuge at 4700× *g* for 5 min before being re-suspended in 150 μL deionized water Re-centrifuge at 4700× *g* for 5 min	**UF**: Concentrate the supernatant by centrifugal ultrafiltration units Amicon^®^ Ultra-15 Centrifugal Filter Unit by centrifugation for 15 min at 3500× *g* Repeat twice centrifugation (~450 μL of the concentrated) RNA extraction: the NucleoSpin ™RNA Virus kit RNA analyses: by OneStep RT-qPCR using Luna Universal Probe One-Step RT-qPCR Kit or LightCycler^®^Multiplex RNA Virus Master and the CFX96 Real-Time System, with a C1000 Touch Thermal Cycler	[49]

Volume: Initial volume; N.A.: not available; N.D.: not detected; COS: colonic organoids; the preconditioned sample; UF: Ultrafiltration; WW: wastewater; MH: manhole; Pumping station. ^A^ Samples comprised of 3 grab samples taken from influent of WWTP, influent of UASB process after mechanical treatment, effluent of the UASB process, aeration tank and effluent of WWTP all in India; ^B^ 24 h composite WW samples from influent and effluent of primary settling tank, effluents of secondary settling tanks from Ourense WWTP in Spain; ^C^ Samples from influent of 7 WWTPs and samples from 2 manholes nearby pandemic hospitals in İstanbul, Republic of Turkey; PBS: phosphate buffer saline. ^D^ Water/wastewater composite and grabs samples 5 sites in Southern Nevada (3 WWTPs, an untreated surface water and finished drinking waters from 3 treatment facilities).

**Table 2:** The protocols applied to wastewater and sewage sludge samples for detecting and quantifying SARS-CoV-2.

Sample	Concentration Method	RNA Extraction	PCR Assays	Ref
24 h composite samples of raw sewage from urban WWTP in Massachusetts, USA	**PEG precipitation** Pasteurize in a 60 °C water bath for 90 min Filter through a 0.2 μm membrane Mix the filtrate with PEG 6000 (a final concentration of 8% (*w v*^−1^) and NaCl to 0.3 M Dissolve the chemical by about 15 min Centrifuge at 12,000× *g* for 2 h Discard the PEG-containing supernatant Re-suspend the pellet 1.5 mL TRIzol reagent	**TRIzol-chloroform method** Mix the re-suspended samples with 300 μL chloroform for 1 min Incubate for 5 min at room temperature Centrifuge at 16,000× *g* at 4 °C for 15 min Transfer aqueous phase to a new 1.5 mL tube Mix with an equal volume of isopropanol Centrifuge at 16,000× *g* for 10 min Discard the supernatant, wash twice the pellet with 75% ethanol, Recover RNA using 30 μL of diethyl pyrocarbonate (DEPC) water	A Bio-Rad CFX96 real-time (RT) PCR detection system PCR: TaqMan Fast Advanced master mix, CDC N1, N2, and N3 primer-probes (IDT); and cDNA as a template	[62]
24 h composite wastewater samples from 7 WWTPs in California, USA	According to the protocol of Wu et al. [62]	According to the protocol of Wu et al. [62]	N.A.	[62]
Grab samples from influent of WWTP in Neuquen city, Argentina	**PEG precipitation** Adjust pH to 6.5–7.2, Add PEG 6000 (10% *w v*^−1^) and NaCl (0.3 M) Stir for 2 h at 4 °C Centrifuge at 10,000× *g* for 25 min at 4 °C Discard the suspension, suspend the pellet in 1 mL PBS (pH 7.2), adjust pH to 8.0, incubate at room temp. for 1 h with occasional agitation, centrifuge the suspension at 10,000× *g* for 20 min, store at −70 °C	The commercial kit Direct-zol RNA Miniprep™	One-step real time RT-PCR assay Target regions of the SARS-CoV-2 nucleocapsid gene (N1 and N2) by TaqMan probe	[96]
Grab samples from influent of WWTP in Neuquen city, Argentina	**Aluminum-driven flocculation [14] Method** Add 1:100 *v v*^−1^ of 9% PAC Adjust pH to 6.0, gently agitate for 30 min at room temp. Centrifuge at 1700× *g* for 20 min Re-suspended pellets into 10 mL of 3% beef extract (pH 7.4), shack for 20 min at 80 rpm Centrifuge at 1900× *g* for 30 min Re-suspended the pellet in 1mL of PBS, store at−70 °C	The commercial kit Direct-zol RNA Miniprep™	One-step real time RT-PCR assay Target regions of the SARS-CoV-2 nucleocapsid gene (N1 and N2) by TaqMan probe	[96]
Influent, secondary and tertiary treated effluent water samples in the Region of Murcia, Spain	**Aluminum-driven flocculation** **As explained in [96]**	The Nucleo-Spin RNA virus kit	TaqMan real-time RT-PCR (RT-qPCR) on LightCycler 480 instrument	[14]
Grab samples from influent of 2 WWTPs and a manhole in a metropolitan region in Japan.	Centrifuge the sample (400) 3000 rpm (1840× *g*) for 30 min **PEG precipitation** According to the protocol of Wu et al. [62] **ENMA** [62,97] Filter with a pore size of 1.0 μm and 0.45 μM membrane, adjust pH to 3.5 using 0.5 N HCl, elute the adsorbed viruses with 3% beef extract solution **UF**: As explained in Table 1	A RNeasy Microbiome kit^®^ For sediment extraction: The RNeasy PowerSoil kit^®^	The One Step PrimeScript III RT-qPCR Mix™ for the NIID_N2 assay The SARS-CoV-2 Direct Detection RT-qPCR Kit™ for the CDC_N1N2 assay.	[85]
Grab samples (after the decantation before the activated sludge process) from WWTP and from suspected COVID-19 patients hospitalized in the local university hospital (60%) and from retirement home residents (40%)	**PEG precipitation** Dissolve 3 g beef extract powder, 3 g NaCl, and 0.37 g glycine in 100 mL of sample, Add 20 g of PEG 6000 to the mixture Stir gently at 4 °C for 2 h, store at 4 °C *overnight* Centrifuge at 4500× *g*, 4 °C for 45 min. re-suspended the pellet in deionised water, add 10 mL NucliSENS^®^ lysis buffer, incubate for 10 min at room temperature **UF** Centrifuge 2 volumes of 50 mL at 1500× *g* for 15 min using MWCO of 100 kD	Transfer the sample in lysis buffer in a 50 mL conical tube, containing 4 g of a mixture of high-vacuum silicon grease (Dow Corning^®^) and silicon dioxide (Sigma) (90:10 *w/w*) Add 15 mL phenol-chloroform-isoamyl alcohol Stir 15 s by hand, centrifuge at 3500× *g* for 5 min Recover the hydrophile supernatant (15 mL) Use 70 μL of magnetic silica beads and the NucliSENS^®^ easyMAG™ platform to extract RNA Elute the extracted in 100 μL of elution buffer Remove residual environmental inhibitors using OneStep PCR Inhibitor Removal kit™,store at −80 °C	Real-time RT-PCR and RT-digital droplet PCR (RT-ddPCR) For the RdRp_IP4, E, and VTB4-Fph GGII sets, quantification using an RNA UltraSens™ One-Step Quantitative RT-PCR system (Applied Biosystems™) StepOnePlus Real-Time PCR System (Applied Biosystems™)	[87]
A grab sample from WWTP in Niigata Prefecture, composite samples WWTPs in Kanagawa Prefecture and Tokyo in Japan	**Pretreatment**: Centrifuge at 3500× *g* for 5 min **PEG precipitation**: using PEG8000 and NaCl (to final concentrations of 10% and 1 M), overnight mixing at 4 °C **UF**: Centrifuge at 3500× *g* for 20min to filter (30 kDa) **Electronegative membrane vortex (EMV)**: Filter of raw sewage (50 mL) inoculated with 500 μL of 2.5 M MgCl_2_ through 0.45 μm membrane by vacuum aspiration.	Spin column-based nucleic acid purification using QIAamp Viral RNA Mini Kit™ Acid guanidinium thiocyanate–phenol– chloroform extraction according to the protocol of Chomczynski and Sacchi [98] using TRIzol LS reagent	qPCR:TaqMan™ Gene Expression Master Mix Specific forward primers/reverse primers, and TaqMan Probe	[88]
24 h composite samples Influent and effluent of 11 WWTPs, MH, PSs	Pasteurize in a 60 °C water bath for 90 min Filter through 0.22 μm polyethersulfone filter **PEG precipitation**: According to Wu et al. [99] **Ultrafiltration**: using MWCO of 30 kDa	ABIOpure Viral DNA/RNA Extraction kit Ultrafiltration columns/RNA extraction kit PEG/TRIzol extraction	RT-qPCR using GENESIG COVID-19 kit	[86]
24 h composite WWTPs in Spain	**PEG precipitation**: with 20% PEG 6000; re-suspend the pellet in 3 mL of PBS, pH 7.4	NucliSENS^®^ miniMAG^®^ extraction system	One-step RT-qPCR assays; UltraSense One-step Quantitative RT-PCR System™	[59]
Grab sample seeding with gamma irradiated (5 × 10^6^ RADs) SARS-CoV-2, PEDV and MgV	Add 25 mL of TGEB pH 9.5 to the seeded sample Incubated at 300 rpm for 2 h at 4 °C. Centrifuge at 2500× *g* for 10 min at 4 °C **PEG Precipitation**: with PEG 8000 and NaCl (20% + 0.3 M) in agitation overnight at 4 °C, centrifuge at 3500× *g* for 30 min at 4 °C, re-suspend the pellet in PBS, store at −80 °C **Aluminum-driven flocculation** According to the protocol of Randazzo et al. [14]	Manual column-based commercial kit Nucleospin RNA virus Kit™ according to the manufacturer’s protocol together with an initial pre-treatment step with Plant RNA Isolation Aid [14] An automated instrument relying on magnetic beads for nucleic acid purification, Maxwell^®^ RSC Instrument used for automated nucleic acid isolation using the Maxwell RSC Pure Food GMO and authentication kit	RT-qPCR using One Step Prime Script™ RT-PCR Kit	[14]
Grab samples influent and UASB effluent of Old Pirana WWTP in Ahmedabad, in India	Centrifuge at 4500× *g* for 30min Filtrate through 0.22 μm membrane filters **PEG precipitation**: with PEG 9000 (80 g L^−1^) and NaCl (17.5 g L^−1^), incubating overnight at 17 °C, at 100 rpm; centrifuge the mixture at 13,000× *g* for 90 min	Mix 10 μL MS2 phage, 20 μL Proteinase K (20 mg mL^−1^) solution and 600 μL of RAV1 buffer containing carrier RNA with the concentrated viral particles (200 μL) and follow steps instructed in the product manual of NucleoSpin^®^ RNA Virus kit	The detection of ORF1ab, N gene and S gene of SARS-CoV-2 and MS2 (internal process control) by RT-PCR using TaqPath™ Covid-19 RT-PCR Kit Real Time PCR system	[25]
^A^ Mixture comprised of 3 grab samples (Table 1)	**Centrifuge/PEG precipitation as described by [25]** Centrifuge at 4500× *g* for 30 min and filtrate (0.22 μm) **UF**: (30 times) using the filtrate using the 96 well filter plate with a capacity to filter less than 10 kDa molecules	Mix 10 μL MS2 phage, 20 μL Proteinase K (20 mg/mL) solution and 600 μL of RAV1 buffer containing carrier RNA with the concentrated viral particles (200 μL) and follow steps instructed in the product manual of NucleoSpin^®^ RNA Virus kit	The detection of ORF1ab, N gene and S gene of SARS-CoV-2 and MS2 (internal process control) by RT-PCR using TaqPath™ Covid-19 RT-PCR Kit	[82]
Samples from 7 WWTPs and samples from 2 MHs nearby pandemic hospitals in İstanbul, Turkey	Centrifugation at 3000× *g* for 45 min **PEG precipitation**: with PEG 8000 (10% *w v*^−1^) and NaCl (0.3 M), incubating overnight at 4 °C, at 60 rpm; centrifuge the mixture at 5700× *g* for 2 h at 4 °C, store at −80 °C	QIAmp cador Pathogen Mini Kit	RT-qPCR QuantiNova Pathogen + IC kit Bio-Rad CFX96 thermal cycler™	[66]
Primary sludge and the waste activated sludge samples from 7 WWTPs in İstanbul, Turkey	**Shake at 100 rpm, at 4 °C for 30 min to transfer viruses into liquid phase** Centrifugation at 7471× *g* for 30 min, sequentially filter supernatant through a 0.45 and 0.2 μm membranes **PEG precipitation: as described [66]**	Extract of 1 mL virus concentrate with Roche MagNA pure LC total nucleic acid isolation kit™ using Roche MagNA pure LC system™ in accordance with the manufacturer’s protocols Quantify the RNA with Thermo NanoDrop 2000c™	Real time ready RNA virus Master™ contained 0.8 nM of forward primer and reverse primer, 0.25 nM probe and 5 μL of template RNA.	[99]
Grab samples from WWTP in Ishikawa prefecture and WWTPs in Toyama prefecture, in Japan	Centrifuge the sample at 5000× *g* for 5 min **PEG precipitation**: with PEG 8000 and NaCl (10% and 1 M) incubating the mixture overnight on a shaker at 4 °C, centrifuge at 10,000× *g* for 30 min, re-suspend the pellet in phosphate buffer	A 60 μL of RNA extract, in accordance with the manufacturer’s instructions using a QIAamp Viral RNA Mini Kit^®^	TaqMan-based qRT-PCR assays One Step PrimeScript™ RT-PCR Kit™ RT-nested PCR assays	[100]
24 h composites, wastewater samples: influent and effluent of primary settling tank, effluents of secondary settling tanks; sludge samples; primary and secondary settling tanks, thickener, thermal hydrolysis (Spain)	Centrifuge at 4500× *g* for 30min **UF**: with a capacity to filter less than 10 kDa molecules by filtration using Amicon 15 mL 10 K centrifugal device, buffer pH to7.4 with PBS **PEG precipitation for the sludge samples**: Add glycine buffer (1:8 (*v v*)) to the sludge, incubate the mixture at 4°C for 2 h, centrifuge at 8000× *g* for 30 min, filter through a 0.45 μm polyethersulfone, precipitate by adding 1:5 (*v v*^−1^) of PEG 8000 (80g/L) and NaCl (17.5 g/L), shack at 150 rpm overnight at 4 °C, centrifuge at 13,000× *g* for 90 min, re-suspended in PBS buffer pH 7.4, store at −80 °C	MicrolabStarlet IVD™ using the STARMag 96 × 4 Universal Cartridge Kit™ according to manufacturer specifications	One-step multiplex RT-qPCR Allplex system™ 2019-nCoV For the RT-PCR, the CFX96 system™	[90]
24 h composite samples from 5 WWTPs in Milan, Turin, and Bologna, Italy	Pasteurize in a 60 °C water bath for 90 min Centrifuge at 1200× *g* for 30 min **Two-phase (PEG-dextran) separation method** Pool the supernatants in a 1 L Erlenmeyer flask and keep the pellets (P1) at 4 °C, adjust the pH of the supernatant to pH 7 –7.5, add 19.8 mL of 22% dextran, 143.5 mL 29% PEG 6000, and 17.5 mL 5N NaCl to 250 mL of supernatant, agitate for 30 min at 4 °C, pour the mixture a sterile conical separation funnel, leave overnight at 4 °C, collect the entire lower layer (S1), re-suspend the pellet (P1) into S1, extract with 20% volume of chloroform by shaking vigorously for 10 min, centrifuge 1200× *g* for 10 min	The NucliSENS miniMAG™ Semi-automated extraction system with magnetic silica	Real-time RT-qPCR assays targeting the E gene of the SARS Betacoronavirus and the RdRp gene of SARS-CoV-2 A newly developed real-time RT-(q)PCR designed using the Primer3 Software targeting the ORF1ab region (nsp14; 3′-to-5′ exonuclease) of the SARS-CoV-2 genome	[58]
24 h composite post-grit chamber influent solids and primary clarified sludge samples from the City of Ottawa’s Robert O. Pickard Environmental Centre, Ontario, and the City of Gatineau, Quebec, WRRFs in Canada	After settling at 4 °C for an hour, decant the supernatant Filter through 1.5 μm glass fiber filter and 0.45 μm GF6 mixed cellulose ester (MCE) filter, collect eluate fraction by passing 32 mL of elution buffer (0.05 M KH_2_PO_4_, 1.0 M NaCl, 0.1% (*v v*^−1^) Triton X-100, pH 9.2) through the spent filters. **PEG Precipitation**: with PEG 8000 (80 g/L and 0.3M NaCl (pH 7.3), agitating at 4 °C and 160 rpm for 12–17 h hours, centrifuge at 10,000× *g* for 45 min at 4 °C, decant the supernatant samples, centrifuge at 10,000× *g* for 10 min, decant the remaining supernatant, transfer a new RNase-free centrifuge tube, store at −80 °C until RNA extraction	Lysis buffer/TRIzol LS extraction Analysis with The RNeasy Power Microbiome^®^ Add 200 mg of sample pellet in place of 200 μL of liquid sample; add lysis buffer/TRIzol LS reagent to maximize lysis of cells/virion encapsulated fragments and protect RNA prior to vortexing and centrifugation; retain the resulting aqueous phase of the lysis procedure, processed as per the recommended protocol including the on-column enzymatic DNA removal step	Singleplex, probe-based, one-step RT-qPCR Singleplex, probe-based, one-step RT-ddPCR	[64]
Grab samples from Quito’s river receiving untreated sewage	**Skim milk flocculation** Prepare the pre-flocculated skimmed-milk solution (PRSMS) (1% *w v*^−1^) [101] Adjust pH to 3.5, add PRSMS of 10 mL L^−1^ to the supernatant Stir slowly at room temp for 8 h Precipitate by centrifugation at 8000× *g* for 40 min Carefully remove the supernatants Re-suspend the pellet in 10 mL of PBS (pH 7.2) Store at −70 °C.	AccuPrep^®^ Universal RNA Extraction Kit	CFX96 Real-Time detection system using TaqMan™ Fast Virus 1-Step Master Mix and the RTqPCR diagnostic panel assays for N1 and N2 regions of N gene	[72]
Composite samples, PS station and 2 WWTPs in Canada	Adjust pH to ~3.5 to 4 (2.0 N HCl) **ENMF**: Pass the sample (100–200 mL) through 0.45-μm-pore-size, 90-mm-diameter electronegative membrane	Use a 5 mL bead tube from RNeasy PowerWater Kit to accommodate the electronegative homogenize the samples ranging from 3 × 20 s at 8000 rpm at a 10 s interval membrane	RT-qPCR assays using a Bio-Rad CFX96 thermal cycler	[82]

WWTP: Wastewater treatment plant; PEG: polyethylene glycol: N.D.: not detected; LOD: detection limit; PAC: aluminum poly chloride; TGEB: tris glycine-beef extract buffer; EB: elution buffer containing 0.2 g/L of sodium polyphosphate, 0.3 g/L of C_10_H_13_N_2_O_8_Na_3_.3H_2_O; UF: ultrafiltration; ENMS: electronegative membrane adsorption; PBS: phosphate buffer solution; MWCO: molecular weight cutoff. ^A^ Samples comprised of 3 grab samples taken from influent of WWTP, influent of UASB process after mechanical treatment, effluent of the UASB process, aeration tank and effluent of WWTP all in India.

## Data Availability

The authors confirm that the data supporting the findings of this study are available within the article.
